# Natriuretic Peptides and Soluble ST2 Improve Echocardiographic and Invasive Long-Term Survival Prediction in Patients Evaluated for Diastolic Dysfunction

**DOI:** 10.3390/ijms26083713

**Published:** 2025-04-14

**Authors:** Horațiu Suciu, Paul-Adrian Călburean, Adina Huțanu, Mădălina Oprica, Diana Roxana Opriș, Anda-Cristina Scurtu, Alexandru Stan, David Aniței, Klara Brînzaniuc, László Hadadi, Marius Harpa

**Affiliations:** 1 Department of Surgery IV, George Emil Palade University of Medicine, Pharmacy, Science and Technology of Targu Mures, 540142 Targu Mures, Romania; 2 Department of Cardiovascular Surgery, Emergency Institute for Cardiovascular Diseases and Transplantation Targu Mures, 540136 Targu Mures, Romania; 3 Department of Biostatistics and Medical Informatics, George Emil Palade University of Medicine, Pharmacy, Science and Technology of Targu Mures, 540142 Targu Mures, Romania; 4 Department of Laboratory Medicine, George Emil Palade University of Medicine, Pharmacy, Science and Technology of Targu Mures, 540142 Targu Mures, Romania; 5 Centre for Advanced Medical and Pharmaceutical Research, George Emil Palade University of Medicine, Pharmacy, Science and Technology of Targu Mures, 540142 Targu Mures, Romania; 6 Doctoral School, George Emil Palade University of Medicine, Pharmacy, Science and Technology of Targu Mures, 540142 Targu Mures, Romania; 7 Department of Interventional Cardiology, Emergency Institute for Cardiovascular Diseases and Transplantation Targu Mures, 540136 Targu Mures, Romania; 8 Department of Anatomy and Embryology, George Emil Palade University of Medicine, Pharmacy, Science and Technology of Targu Mures, 540142 Targu Mures, Romania

**Keywords:** mid-regional pro-atrial natriuretic peptide, B-type natriuretic peptide, N-terminal prohormone B-type natriuretic peptide, soluble ST2, galectin-3, mid-regional pro-adrenomedullin

## Abstract

This study aimed to investigate the impact of long-term survival on cardiac serum biomarkers such as natriuretic peptides (mid-regional pro-atrial natriuretic peptide [MR-proANP], B-type natriuretic peptide [BNP], N-terminal prohormone BNP [NT-proBNP]), soluble ST2 (sST2), galectin-3 and mid-regional pro-adrenomedullin (MR-proAMD). Consecutive patients hospitalized in a tertiary center, undergoing echocardiographic and invasive left cardiac catheterization for diastolic dysfunction assessment were prospectively included in this study. Cardiac biomarkers were determined from pre-procedural peripheral venous blood samples. A total of 110 patients were included, with a median follow-up of 1.66 (1.23–2.16) years during which 16 (14.5%) patients died. A total of 45.4% (50) of patients had diastolic dysfunction. In the univariate Cox regression, long-term survival was predicted by BNP (*p* < 0.0001, HR = 0.39 [0.20–0.53]), NT-proBNP (*p* < 0.0001, HR = 0.40 [0.22–0.55]), MR-proANP (*p* = 0.001, HR = 0.30 [0.11–0.46]), sST2 (*p* < 0.0001, HR = 0.47 [0.30–0.60]), but not with MR-proAMD (*p* = 0.77) or galectin-3 (*p* = 0.76). In the final stepwise multivariable Cox regression non-invasive and invasive models, NT-proBNP and sST2 remained independent predictors of survival. Natriuretic peptides (BNP and NT-proBNP) and sST2 were predictors of long-term survival, while MR-proANP, MR-proADM and galectin-3 did not have predictive values. NT-proBNP and sST2 improved survival prediction in both a non-invasive scenario (including clinical, serum and echocardiographic parameters) and an invasive clinical scenario (including left heart catheterization parameters). The sST2 pathway could provide a target for therapeutic intervention.

## 1. Introduction

Heart failure (HF) remains a major public health challenge, as it has high morbidity and mortality, despite significant advances in treatment [1]. Accurate prediction of long-term survival in HF patients is essential for guiding clinical management and optimizing therapeutic strategies. However, traditional risk scores based on clinical parameters and conventional imaging techniques often fall short of fully capturing the complex pathophysiology of HF. As such, there is a growing interest in the use of circulating biomarkers that can provide additional prognostic information [1].

Natriuretic peptides, such as B-type natriuretic peptide (BNP) and its inactive fragment, N-terminal pro–B-type natriuretic peptide (NT-proBNP), are well-established markers of cardiac wall stress [2]. These peptides are released in response to increased ventricular pressure and volume overload, and they have been consistently associated with worse outcomes in HF [2]. Elevated levels of BNP and NT-proBNP not only reflect hemodynamic burden but also correlate with structural and functional cardiac abnormalities, making them indispensable tools in both diagnosis and prognosis [1].

Beyond natriuretic peptides, emerging biomarkers offer insights into additional pathophysiological processes such as inflammation, fibrosis and remodeling, which also contribute to HF progression [3]. The soluble suppression of tumorigenicity-2 (sST2) is one such novel biomarker that has attracted considerable attention over the past decade. sST2, a member of the interleukin-1 receptor family, is released in response to mechanical strains and inflammatory stimuli [4]. Multiple studies have demonstrated that sST2 independently predicts mortality and HF hospitalization, even after adjusting for established markers like NT-proBNP [5]. Its incremental prognostic value underscores the benefit of a multi-biomarker approach, as sST2 captures aspects of myocardial stress and fibrotic remodeling that natriuretic peptides may not fully reflect.

Other biomarkers, such as mid-regional pro-atrial natriuretic peptide (MR-proANP), galectin-3 and mid-regional pro-adrenomedullin (MR-proADM), have also been investigated for their roles in HF [3,6]. While MR-proANP shares similarities with BNP and NT-proBNP in reflecting cardiac stretch, galectin-3 and MR-proADM are more closely linked to inflammation and vascular function, respectively. However, it is unclear whether such biomarkers could provide prognostic information.

This study aimed to investigate the impact on long-term survival of cardiac serum biomarkers such as natriuretic peptides (MR-proANP, BNP, NT-proBNP), sST2, galectin-3 and MR-proAMD, and to investigate whether such biomarkers could improve the survival prediction in clinical parameters, echocardiographic parameters and left heart catheterization in patients evaluated for diastolic dysfunction.

## 2. Results

A total of 110 patients were included, of which 75.4% (83) were males, with a mean age of 62.6 ± 12.6 years. The complete baseline characteristics of the studied population are summarized in Table 1. During a median follow-up of 1.66 (1.23–2.17) years, a total of 16 (14.5%) deaths occurred, of which 10 (9.0%) deaths occurred within the first year. Sex, age or body mass index had no impact on survival (Table 1). In univariate Cox regression, only comorbidities such as chronic kidney disease and valvular heart disease affected survival (Table 1). Interestingly, echocardiographic parameters of the systolic function, such as LVEF and stroke volume had no impact on mortality in Cox univariate regression (Table 2). Patients with lower global longitudinal strain, a more sensitive echocardiographic parameter, had higher 1-year mortality and significantly impacting outcomes in univariate Cox regression analysis (Table 2). Echocardiographic presence and grade of diastolic dysfunction had a consistent negative effect on both 1-year survival and longer survival (Table 2). The left atrium reservoir strain, a newer parameter of diastolic dysfunction, was lower in patients who died during follow-up. Increased ventricular and atrium volumes were also associated with impaired survival during follow-up (Table 2).

**Figure 1:**
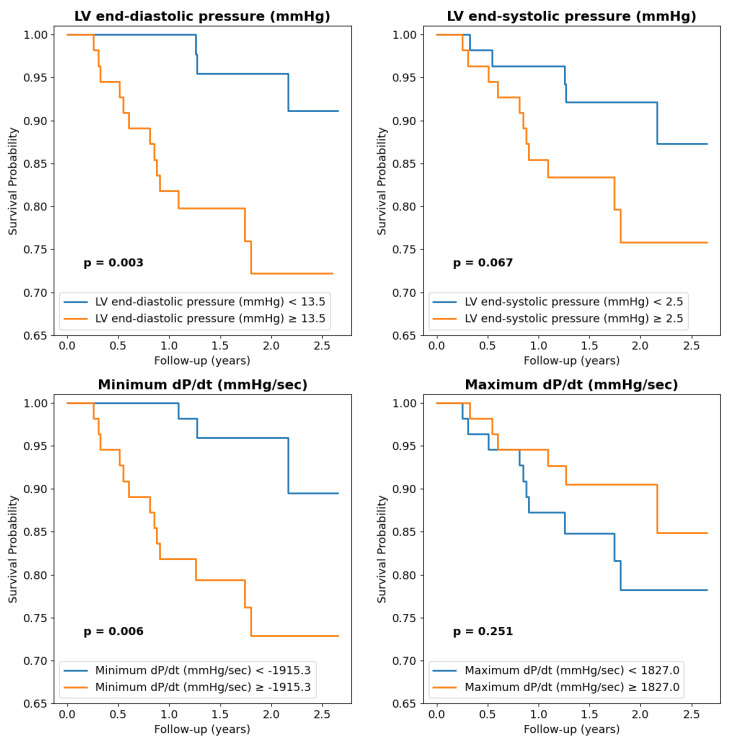

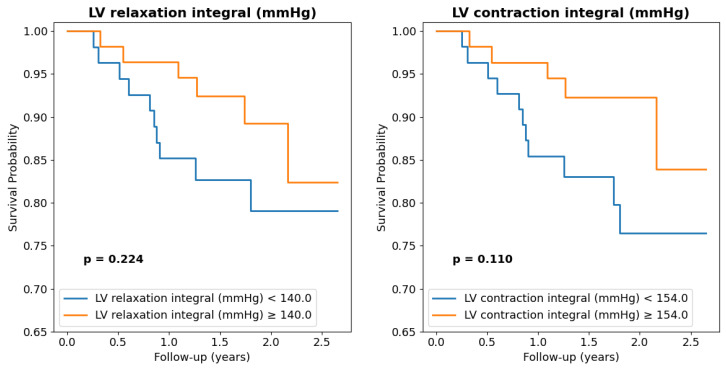
Impact of left heart catheterization-derived parameters on long-term survival. Median values were used as cut-off.

The mean LVEDP was 14.0 ± 8.0 mmHg, and 45.4% (50) of patients had diastolic dysfunction when considering an LVEDP above 15 mmHg as a cut-off. LVEDP was higher in patients with one-year death and a significant negative prognostic in Cox univariate regression. A consistent impact on survival was also documented for other invasive parameters such as the minimum dP/dt or relaxation integral, for which lower values were associated with one-year death and long-term impaired survival (Table 2). In Kaplan–Meier curves, both patients with an LVEDP below the median 13.5 mmHg or with a minimum dP/dt > −1915 mmHg/sec had lower survival (Figure 1).

**Figure 2:**
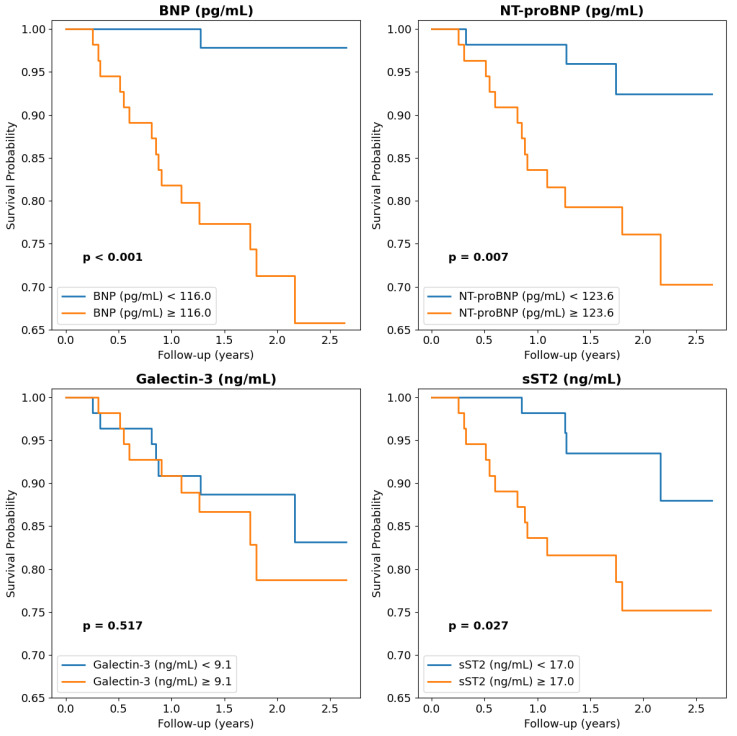

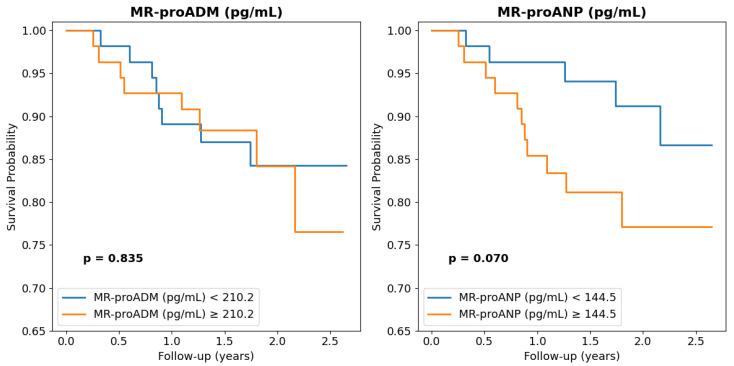
Impact of serum biomarkers on long-term survival. Median values were used as cut-off.

Natriuretic peptides such as NT-proBNP and BNP were significantly higher in patients with one-year death and had a significant negative impact on long-term survival (Table 2). Similarly, in Kaplan–Meier plots, patients with an NT-proBNP or BNP above the median values of 123.6 pg/mL and 110.0 pg/mL, respectively, had lower long-term survival (Figure 2). In contrast, MR-proANP, another natriuretic peptide, had no impact on survival. sST2 was significantly higher in patients with one-year death and had a significant negative impact on long-term survival (Table 2). Similarly, in Kaplan–Meier plots, patients with an sST2 above the median value of 17.0 ng/mL had a lower long-term survival (Figure 2). Surprisingly, MR-proAMD and galectin-3 had no impact on survival in any analysis (Table 2 and Figure 2).

Multivariate analysis was performed using two clinical scenarios: (1) a non-invasive scenario using clinical parameters, serum biomarkers and echocardiographic parameters, and (2) an invasive scenario, performed by adding parameters measured during left heart catheterization. The two scenarios were considered since left heart catheterization, while being the gold standard in diastolic dysfunction diagnosis, is not a routinely performed investigation, and is associated with higher risk, and thus, is not suitable for screening. In the non-invasive setting, echocardiographic parameters such as an E/E’ ratio above 9.8 and an LVESV above 41 mL/m^2^ were predictors of events, along with an NT-proBNP above 123 pg/mL and an sST2 above 17 ng/mL (Table 3). In the invasive setting, the catheterization-derived parameter minimum dP/dt > −1915 mmHg/sec was a predictor of events, along with an NT-proBNP above 123 pg/mL, an sST2 above 17 ng/mL and an E/E’ ratio above 9.8 (Table 3). The predictive performance for the two models was high, with a C-statistic of 0.786 for the non-invasive model and 0.823 for the invasive model (Table 3).

## 3. Discussion

The main findings of our study can be summarized as follows: (1) natriuretic peptides (BNP and NT-proBNP) and sST2 were predictors of long-term survival, while MR-proANP, MR-proADM and galectin-3 did not have predictive value; (2) echocardiographic parameters such as E/e’ ratio and left atrium strain were predictors of events, while left heart catheterization parameters such as minimum dP/dt and LVEDP were predictors of events; (3) NT-proBNP and sST2 improved survival prediction in both a non-invasive and an invasive clinical scenario.

HF is a complex syndrome with multiple pathological pathways, and no single biomarker captures the full picture. In recent years, attention has turned to multi-biomarker approaches—combining markers of different pathophysiological processes (myocardial stretch, fibrosis, inflammation, remodeling)—to better identify patients at risk [7]. Our study revealed relatively high mortality in diastolic dysfunction, as a total of 16 (14.5%) deaths occurred during a median follow-up of 1.66 years. Indeed, cardiovascular diseases are particularly associated with high mortality [8]. Natriuretic peptides, released in proportion to ventricular wall stress, are the benchmark against which newer biomarkers are compared and are the only biomarkers currently recommended by clinical guidelines for diagnosis [1]. High NT-proBNP levels reflect elevated filling pressures and volume overload, and numerous studies confirm its strong association with adverse outcomes [9,10]. Clinically, NT-proBNP is used to stratify risk, and patients with higher concentrations face a significantly greater likelihood of death or HF hospitalization. For instance, an NT-proBNP-guided strategy in chronic HF can identify patients who might benefit from more aggressive therapy, although the NT-proBNP alone has limitations (e.g., levels can be affected by renal function, age, or obesity). It has become routine to measure NT-proBNP in HF patients, yet researchers have recognized that combining NT-proBNP with complementary markers could improve predictive accuracy [11]. The therapeutic modulation of the natriuretic peptides pathway with angiotensin receptor–neprilysin inhibitors (ANRI) led to a significant outcome reduction in heart failure with reduced ejection fraction [12]. Even more, improved survival with ARNI was also observed in heart failure with mildly reduced or preserved ejection fraction, in which diastolic dysfunction is predominant [13].

Among the emerging biomarkers, the soluble suppression of tumorigenicity-2 (sST2) has shown particular promise in augmenting prognostic models. sST2 is a novel biomarker reflecting myocardial fibrosis and inflammatory stress, representing a different pathway than natriuretic peptides. Over the past decade, a robust evidence base has established sST2 as a powerful independent predictor of survival in HF. Elevated sST2 levels have been linked to worse outcomes across multiple studies and patient cohorts. Notably, sST2 retains prognostic value even after accounting for NT-proBNP and clinical risk factors, indicating additive information [4]. A recent systematic review of 11 studies (over 5000 patients) confirmed that higher sST2 concentrations predict worse long-term outcomes, including all-cause and cardiovascular mortality and HF hospitalization [14]. Combining multiple biomarkers that reflect diverse disease mechanisms can yield a more accurate prognostic assessment than any single marker alone. NT-proBNP and sST2 exemplify this synergy: NT-proBNP tracks hemodynamic load, whereas sST2 captures fibrotic and inflammatory activity—two complementary facets of HF pathophysiology. The Penn Heart Failure Study (over 1100 chronic HF patients) demonstrated that sST2 is a powerful indicator of prognosis and provides moderate improvement in risk stratification when added to conventional biomarkers like BNP or atrial natriuretic peptide [15]. Patients with the highest sST2 levels had a significantly increased risk of death or transplant, even after accounting for natriuretic peptides, especially in non-ischemic HF cohorts [15]. This suggests sST2 captures high-risk features not fully reflected by natriuretic peptide levels. A study examined a panel of NT-proBNP (marker of myocardial stretch), high-sensitivity troponin T (marker of myocyte injury) and sST2 (fibrosis/remodeling). Adding sST2 and troponin to a clinical risk model led to a net reclassification improvement of ~14% for mortality risk [16]. Thus, a significant subset of patients were more correctly stratified when these biomarkers were included, highlighting that the information provided by sST2 and troponin is non-redundant with NT-proBNP [16]. Consistently, Emdin et al. reported that sST2 conferred strong predictive power for all-cause and cardiovascular death and improved risk classification even when NT-proBNP and troponin were already in the model [17]. Our results also suggest that sST2 provides added prognostic value to natriuretic peptides and support targeting the sST2 pathway as a therapeutic target. Indeed, sST2 modulation has been recently proposed as a potential modulating target for heart failure [18,19]. Even more, recent proteomic serum analyses found in vivo sST2 inhibitors that improved symptoms and survival [20]. Interestingly, in a study that investigated the impact of ARNI treatment on serum biomarkers, ARNI also reduced sST2 levels [21]. However, the reduction was small (approximately 5% reduction), and sST2 levels both before and after ARNI treatment remained predictive for impaired survival [21]. These findings suggest that while sST2 is indirectly influenced by cardiovascular drugs, a direct sST2 inhibitor could still lead to additional survival improvement.

Galectin-3 is another fibrosis-related biomarker that has been studied in HF prognostication. However, head-to-head comparisons indicate that sST2 outperforms galectin-3 in predicting outcomes. In a cohort of 876 patients, both sST2 and galectin-3 levels were associated with higher all-cause mortality, but only sST2 predicted cardiovascular death [22]. This suggests galectin-3 may reflect earlier-stage remodeling, whereas sST2 more directly signals advanced cardiac stress with imminent risk. Thus, multi-marker strategies have gravitated toward sST2 (often alongside natriuretic peptides and troponins) as a key component of prognostic models, given its consistency and strength of association with survival [23]. Similarly, our results suggest that galectin-3 may not provide incremental prognostic value to natriuretic peptides and sST2. In a study that investigated the impact of ARNI on serum biomarkers, ARNI did not influence Galectin-3 levels, and Galectin-3 levels both before and after ARNI treatment did not have predictive value for impaired survival [21].

### Study Strengths and Limitations

Our study’s strength is that the patients were evaluated using left heart catheterization, which is the gold standard in diastolic dysfunction assessment. Moreover, we used a wide panel of cardiac biomarkers and relevant echocardiographic parameters. The main limitation of this study is the relatively low number of patients. Although bootstrapped confidence intervals are reliable for internal validation, our findings require external validation. While the authors acknowledge that our multivariable models may be only partially confirmed, the present work should encourage future works incorporating cardiac biomarkers, especially natriuretic peptides and sST2, for assessing long-term patient prognosis.

## 4. Materials and Methods

All patients scheduled for coronary angiography at the Emergency Institute for Cardiovascular Diseases and Transplantation of Târgu Mureş who also agreed to an additional invasive left ventricular pressure measurement were prospectively enrolled. Participants provided informed consent for both the coronary angiography and cardiac catheterization procedures. The exclusion criteria were: (1) age below 18 years, (2) any acute cardiac condition (for example, acute heart failure, acute coronary syndrome, or acute pulmonary embolism), (3) presence of a valvular prosthesis, (4) use of implantable cardiac rhythm devices with active stimulation, or (5) a suboptimal echocardiographic window. Collected data included anthropometric measurements, relevant medical history, clinical status at hospital admission, ongoing chronic medical treatments and routine laboratory parameters. Additionally, transthoracic echocardiography was performed on all patients before left heart catheterization using a commercially available ultrasound system (GE Vivid E95) and processed with proprietary software (EchoPAC version 202, GE Vingmed Ultrasound).

### 4.1. Biomarker Quantification

Peripheral blood samples were collected before the catheterization procedure and serum samples were stored at −80 °C until analysis. BNP was measured by chemiluminescent immunoassay on an Abbott Architect automated analyzer (Abbott Diagnostics, Abbott Park, IL, USA). An enzyme-linked immunosorbent assay (ELISA) was used to determine NT-proBNP (Biomedica, Vienna, Austria), MR-proANP (AssayGenie, Dublin, Ireland), sST2 (Critical Diagnostics Presage, San Diego, CA, USA), galectin-3 (R&D Systems, Minneapolis, MN, USA) and MR-proAMD (AssayGenie, Dublin, Ireland). All ELISA analyses were performed on the Elisa Dynex DSX fully automated Elisa analyzer (DYNEX Technologies, Chantilly, VA, USA).

### 4.2. Left Ventricular End-Diastolic Pressure Measurement

**Figure 3:**
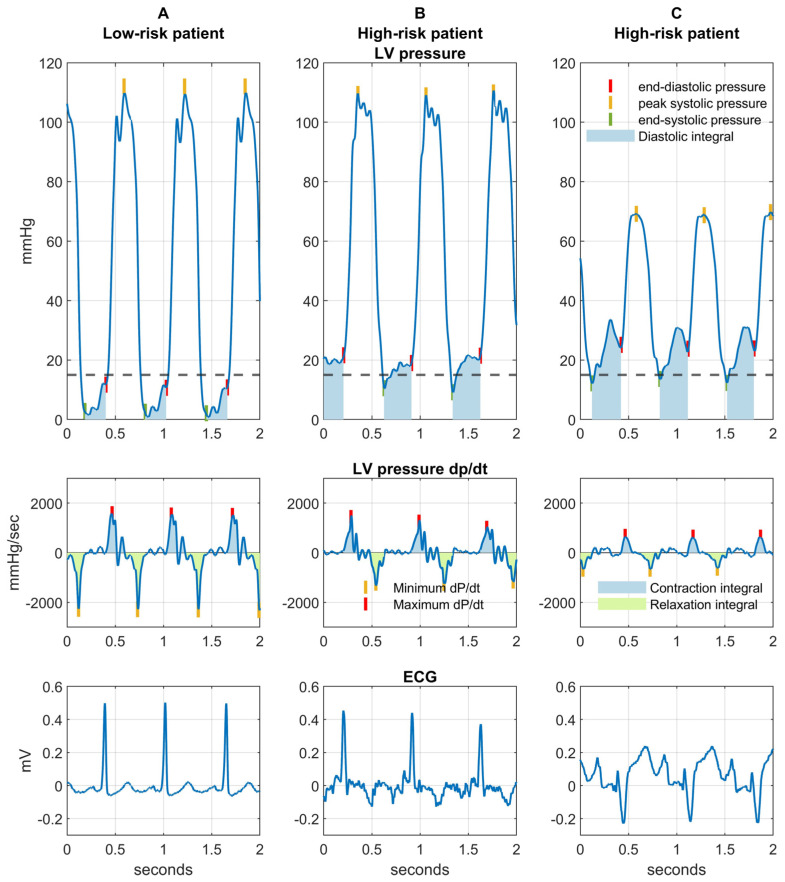
Illustration of left ventricular pressure curve analysis. (**A**)-Low-risk patient with low ejection fraction, normal diastolic function and normal serum biomarkers, but without events during long follow-up. (**B**)-High-risk patient with normal ejection fraction, impaired diastolic function and high serum biomarkers, who died during follow-up. (**C**)-High-risk patient with low ejection fraction, impaired diastolic function and high serum biomarkers, who died during follow-up.

LV pressures were recorded prior to the coronary angiography (i.e., before any contrast injection) using a Siemens Artis zee system (Siemens Healthcare, Forchheim, Germany). A 5-French Judkins right catheter was balanced to the zero level at the mid-axillary line. LV pressures were recorded at end-expiration for five consecutive cardiac cycles. All parameters were averaged over the five consecutive cardiac cycles. Left ventricular diastolic dysfunction was defined as an invasively measured LVEDP elevated above 15 mmHg. Pressure curves were exported and post-processed using MatLab R2023b (MathWorks, Inc., Natick, MA, USA, Figure 3), a software commonly used in biological signal processing [24]. Additional invasively determined variables characterizing left ventricular function were the maximum dP/dt, minimum dP/dt, LV contraction integral (area under the dP/dt curve between end-diastolic and peak systolic pressure), LV relaxation integral (area under the dP/dt curve between peak systolic and end-systolic pressure) and LV diastolic integral (area under the LV pressure curve between end-systolic and end-diastolic pressure, Figure 3).

### 4.3. Statistical Analysis

A significance level α of 0.05 and a 95% confidence interval (CI) were considered. All reported 95% CI were bootstrapped with 1000 samples [25]. Continuous variables were evaluated for normal distribution using the Shapiro–Wilk test. Continuous variables with normal distributions were reported as mean ± standard deviation and compared using the unpaired Student t-test, while continuous variables without normal distributions and discrete variables were reported as median (interquartile range) and compared using the Mann–Whitney U test. Categorical variables were reported as absolute and relative frequencies and were compared using Fisher’s exact test. Multivariable models were constructed using multivariable Cox regression. All multivariable models were constructed in a stepwise forward selection by using the Bayesian information criterion [26]. The predictive performance was analyzed using the C-statistic [27]. Statistical analysis was performed using statsmodels, scipy and scikit-learn libraries implemented in Python version 3.9, while figure plotting was performed using matplotlib library.

## 5. Conclusions

Natriuretic peptides (BNP and NT-proBNP) and sST2 were predictors of long-term survival, while MR-proANP, MR-proADM and galectin-3 did not have predictive value. NT-proBNP and sST2 improved survival prediction in both a non-invasive scenario (including clinical, serum and echocardiographic parameters) and an invasive clinical scenario (including left heart catheterization). The sST2 pathway could provide a target for therapeutic intervention.

## Figures and Tables

**Table 1:** Clinical characteristics of the studied population.

Parameter	1-Year Survival			Long-Term Survival	
	Alive (*n* = 100)	Deceased (*n* = 10)	*p* *	HR	*p* **
Male sex	75 (75%)	8 (80%)	1.00	1.01	0.98
Age (years)	65.9 (56.0–71.7)	59.4 ± 18.3	0.52	0.99	0.68
BMI (kg/m^2^)	29.05 ± 4.51	27.36 ± 3.61	0.25	0.92	0.17
Arterial hypertension	70 (70%)	3 (30%)	0.01	0.43	0.09
Hyperlipidemia	76 (76%)	5 (50%)	0.13	0.56	0.26
Diabetes mellitus	31 (31%)	4 (40%)	0.71	2.10	0.14
Peripheral artery disease	19 (19%)	1 (10%)	0.67	0.59	0.49
Stroke	9 (9%)	0 (0%)	0.60	0.63	0.66
COPD	6 (6%)	1 (10%)	1.00	0.9	0.92
Chronic kidney disease	5 (5%)	3 (30%)	0.02	3.74	0.03
Atrial fibrillation	18 (18%)	5 (50%)	0.03	2.45	0.08
Coronary artery disease	68 (68%)	5 (50%)	0.31	0.44	0.10
History of PCI/CABG	39 (39%)	4 (40%)	1.00	0.91	0.86
History of MI	27 (27%)	2 (20%)	0.72	1.30	0.62
Valvular heart disease	12 (12%)	8 (80%)	0.0005	8.25	0.0001
Dilated cardiomyopathy	21 (21%)	5 (50%)	0.04	2.21	0.12
Concentric hypertrophy	35 (35%)	3 (30%)	1.00	0.86	0.78
QRS duration (ms)	85 (80–100)	121.4 ± 41.12	0.03	1.02	0.01
Creatinine clearance	105.19 ± 30.77	91.47 ± 38.6	0.19	0.99	0.19
LVEF ≤ 40	22 (22%)	5 (50%)	0.06	2.04	0.17
LVEF = 41–49%	15 (15%)	1 (10%)	1.00	0.85	0.82
LVEF ≥ 50%	63 (63%)	4 (40%)	0.20	0.59	0.29
Beta-blocker	78 (78%)	8 (80%)	1.00	1.13	0.84
ACEI/ARB	73 (73%)	3 (30%)	0.009	0.44	0.11
MRA	33 (33%)	8 (80%)	0.007	3.37	0.01
ARNI	16 (16%)	3 (30%)	0.38	1.26	0.71
SGLT2 inhibitors	20 (20%)	5 (50%)	0.04	2.53	0.07
Loop diuretics	31 (31%)	9 (90%)	0.001	6.44	0.001

ACEI—angiotensin-converting enzyme inhibitor; ARB—angiotensin receptor blocker; ARNI—angiotensin receptor/neprilysin inhibitor; BMI—body mass index; CABG—coronary artery bypass graft; COPD—chronic obstructive pulmonary disease; LVEF—left ventricular ejection fraction; MRA—mineralocorticoid receptor blocker; MI—myocardial infarction; PCI—previous coronary intervention; SGLT2—sodium-glucose co-transporter 2. * *p*-value was obtained by comparing patients with and without 1-year mortality. ** *p*-value was obtained by using univariate Cox regression.

**Table 2:** Characteristics of serum biomarkers, echocardiographic and catheterization parameters in the studied population.

Parameter	1-Year Survival			Long-Term Survival	
	Alive (*n* = 100)	Deceased (*n* = 10)	*p* *	HR	*p* **
Serum biomarkers					
MR-proANP (pg/mL)	138 (98–193)	229.2 ± 159.4	0.09	1.00	0.13
BNP (pg/mL)	106 (55–240)	464 (448–531)	0.0001	1.02	0.0001
NT-proBNP (pg/mL)	104 (29–341)	2230 ± 1510	0.00002	1.02	0.0001
sST2 (ng/mL)	16.3 (12.7–21.4)	42.3 ± 27.3	0.0007	1.06	0.0001
Galectin-3 (ng/mL)	9.0 (7.1–11.1)	12.8 ± 8.7	0.25	1.13	0.009
MR-proADM (pg/mL)	216 (126–575)	188 (121–234)	0.33	1.00	0.51
Echocardiographic parameters					
LVEF (%)	50 (41–55)	35 ± 16	0.02	0.97	0.09
Average GLS (%)	−16.4 (−17.9–−11.3)	−9.0 ± 4.6	0.002	1.14	0.007
LVEDV (mL/m^2^)	53.9 (46.6–66.9)	80.0 ± 34.7	0.07	1.01	0.36
LVESV (mL/m^2^)	26.2 (20.9–42.4)	63.4(25.5–91.8)	0.02	1.01	0.004
Stroke volume (mL/m^2^)	26.6 (22.7–31.7)	31.2 ± 10.3	0.33	1.04	0.27
LA volume (mL/m^2^)	35.6 (29.7–43.7)	59.6 ± 17.2	0.0004	1.05	0.0001
LARS (%)	14.9 (10.6–18.3)	7.9 ± 4.0	0.0004	0.89	0.008
RA volume (mL/m^2^)	25.5 (21.5–31.6)	40.4 ± 13.2	0.01	1.04	0.002
MV E/A ratio	8.65 (7.49–10.5)	14.77 ± 5.15	0.0004	1.59	0.005
MV DT (msec)	213.88 ± 52.36	201.61 ± 48.34	0.47	0.99	0.27
Mitral average E/e’	0.85 (0.74–1.45)	1.94 ± 0.84	0.007	1.30	0.0001
Diastolic dysfunction	20 (20%)	8 (80%)	0.0005	5.57	0.0009
Diastolic dysfunction grade	1 (0–1)	2 (2–3)	0.0003	2.21	0.0008
Left ventricular catheterization parameters					
LVEDP (mmHg)	12 (8–19)	20 ± 5	0.01	1.05	0.04
LV end-systolic pressure (mmHg)	2 (−1–6)	9 (7–10)	0.01	1.08	0.06
Minimum dP/dt (mmHg/sec)	−1979 (−2260–−1492)	−1161 ± 389	0.0003	1.02	0.001
Maximum dP/dt (mmHg/sec)	1841 (1373–2179)	1415 ± 737	0.04	1.00	0.06
LV contraction integral (mmHg)	158.0 ± 33.9	120.6 ± 51.5	0.04	0.98	0.03
LV relaxation integral (mmHg)	140.1 ± 27.8	109.4 ± 34.2	0.001	0.98	0.009
LV diastolic integral (mmHg·sec)	22 (16–32.4)	26.1 (23.5–32.7)	0.15	1.01	0.73

DT—deceleration time; GLS—global longitudinal strain; LA—left atrium; LARS—left atrium reservoir strain; LVEDP—left ventricular end-diastolic pressure; LVEDV—left ventricular end-diastolic volume; LVESV—left ventricular end-systolic volume; LVEF—left ventricular ejection fraction; MV—mitral valve; RA—right atrium. * *p*-value was obtained by comparing patients with and without 1-year mortality. ** *p*-value was obtained by using univariate Cox regression.

**Table 3:** Multivariable Cox models for long-term survival prediction in non-invasive and invasive clinical scenarios.

Parameter *	HR (95% CI)	*p*	C-Statistic
Non-invasive setting (clinical and echocardiographic parameters)			
E/E’ ratio > 9.8	2.02 (1.05–4.10)	0.02	0.786
NT-proBNP > 123 pg/mL	2.25 (1.09–3.41)	0.005	
sST2 > 17 ng/mL	1.58 (0.47–2.69)	0.01	
LVESV > 41 mL/m^2^	1.15 (1.01–2.31)	0.04	
Invasive setting (clinical, echocardiographic and catheterization parameters)			
Minimum dP/dt > −1915 mmHg/s	2.13 (1.12–4.21)	0.03	0.823
NT-proBNP > 123 pg/mL	2.54 (1.37–3.72)	0.005	
sST2 > 17 ng/mL	1.50 (0.34–2.60)	0.01	
E/E’ ratio > 9.8	1.76 (1.62–2.92)	0.02	

CI—confidence interval; HR—hazard ratio; LVESV—left ventricle end-systolic volume. * Median values were used as cut-off.

## Data Availability

Due to national and EU regulations, particularly the General Data Protection Regulation (GDPR), the data used in this study cannot be made publicly available and shared with the wider research community. However, the data can be shared by the corresponding author for use in secure environments upon reasonable request.
